# A Combined Approach to Cartographic Displacement for Buildings Based on Skeleton and Improved Elastic Beam Algorithm

**DOI:** 10.1371/journal.pone.0113953

**Published:** 2014-12-03

**Authors:** Yuangang Liu, Qingsheng Guo, Yageng Sun, Xiaoya Ma

**Affiliations:** 1 School of Resource and Environmental Science, Wuhan University, Wuhan, China; 2 State Key Laboratory of Information Engineering in Surveying Mapping and Remote Sensing, Wuhan University, Wuhan, China; 3 School of Geoscience, Yangtze University, Wuhan, China; Xiamen University, China

## Abstract

Scale reduction from source to target maps inevitably leads to conflicts of map symbols in cartography and geographic information systems (GIS). Displacement is one of the most important map generalization operators and it can be used to resolve the problems that arise from conflict among two or more map objects. In this paper, we propose a combined approach based on constraint Delaunay triangulation (CDT) skeleton and improved elastic beam algorithm for automated building displacement. In this approach, map data sets are first partitioned. Then the displacement operation is conducted in each partition as a cyclic and iterative process of conflict detection and resolution. In the iteration, the skeleton of the gap spaces is extracted using CDT. It then serves as an enhanced data model to detect conflicts and construct the proximity graph. Then, the proximity graph is adjusted using local grouping information. Under the action of forces derived from the detected conflicts, the proximity graph is deformed using the improved elastic beam algorithm. In this way, buildings are displaced to find an optimal compromise between related cartographic constraints. To validate this approach, two topographic map data sets (i.e., urban and suburban areas) were tested. The results were reasonable with respect to each constraint when the density of the map was not extremely high. In summary, the improvements include (1) an automated parameter-setting method for elastic beams, (2) explicit enforcement regarding the positional accuracy constraint, added by introducing drag forces, (3) preservation of local building groups through displacement over an adjusted proximity graph, and (4) an iterative strategy that is more likely to resolve the proximity conflicts than the one used in the existing elastic beam algorithm.

## Introduction

Map generalization is an abstraction process that aims to represent geographic information according to the scale and purpose of the map [1], [2]. Nowadays, the widespread use of geographic information in computers in the context of geographic information systems (GIS) has increased the demand for automation of map generalization [3]. Scale reduction from source to target maps inevitably leads to conflicts in map symbols, in which map objects overlap or become too dense to be clearly distinguished [4]. To remove these conflicts, several generalization operators should be used, such as displacement, deletion, exaggeration, aggregation, and typification [5]. Among these operators, displacement is one of the most important contextual operators used to resolve the problems that arise among two or more conflicting map objects. Although the cartography and GIS communities have reported various approaches to automated displacement, displacement operations are still conducted manually or interactively by cartographic experts in most national mapping agencies (NMAs) [6]. Finding better automated displacement approaches to automated map generalization is necessary.

This article proposes an automated building displacement approach based on the combined use of the constrained Delaunay triangulation (CDT) skeleton and the elastic beam algorithm. In the approach, the detection and resolution of conflicts are controlled by a set of cartographic constraints, such as legibility, positional accuracy, and global and local constraints of spatial relationships and patterns.

The major challenge posed by the displacement problem is preserving the spatial relationships and patterns of a set of map objects during the displacement process [4], [7]. An improved elastic beam algorithm was used to solve this problem. In previous studies [7], [8], the displacement results of the elastic beam algorithm are cartographically pleasing. However, the following issues require further discussion: (1) the results of the displacement are affected by the algorithm parameters of the model, but a feasible method for automated setting of these parameters is not yet available; (2) the external energy does not always sufficiently compensate the increasing internal energy; so the required minimum separation distance between map objects cannot be ensured; (3) constraints of local spatial relationships and patterns and positional accuracy are not sufficiently enforced although they are important for the generalization of large- and medium-scale maps. Therefore, we attempt to present several improvements to compensate for these deficiencies.

In addition, displacement is a contextual operator; the process should be supported by enhanced data models [9]. CDT is an ideal tool for proximity modeling and analysis [10], [11]. CDT allows extraction of the skeleton of the map space. This process has been used in many studies involving map generalization [12]–[17]. The CDT skeleton of the map space was here used to facilitate the proximity conflict detection process and to provide essential spatial contextual information for the displacement process.

The remainder of this article is structured as follows. Section 2 briefly reviews previous studies on automated displacement in map generalization. Section 3 clarifies related constraints, conflicts, and measures in building displacement problem and describes the displacement mechanism to be implemented in this approach. Section 4 presents the details of the methods included in the proposed approach. Experimental results are discussed in Section 5 to illustrate the feasibility of the implemented approach. Section 6 concludes this study.

## Literature Review

Generally, automated displacement approaches in map generalization can be classified into two types, namely, sequential and optimization approaches [7], [8], [18]. For sequential approaches, map objects are displaced one by one, step by step, without overall evaluation of the result. Optimization approaches account for the geometries of all map objects and resolve all conflicts simultaneously through either a combinatorial or function approach.

### Sequential approaches

In the early 1970 s, researchers in Germany focused on the basic sequential approaches [19]. The most representative approach may be the equation system for displacement vectors proposed by Lichtner [20]. Using similar ideas as a foundation, Nickerson achieved the displacement of line features by using buffering to detect proximity conflict and a triangle filter to smooth the displacement [21]. Mackaness proposed a modified proportional radial displacement in which displacement occurred in a radial arrangement about the selected center of the conflicting point cluster [22].

Unlike the aforementioned basic sequential approaches, more complex sequential approaches use auxiliary data structures and enhanced data models to support the displacement operation. These include Delaunay triangulation (DT) [1], Voronoi diagram (VD) [23], [24], “displacement mountain” of raster data format [25], and vector-raster hybrid data structure [26]. These studies indicated that identifying conflicts and extracting the contextual information of the involved map objects are necessary for successful use of the displacement algorithm.

### Optimization approaches

Inspired by the ideas of point feature map labeling, researchers have proposed several combinatorial approaches [4], [6], [27], [28] in which the new locations of map objects are calculated using trials. These trials can be realized based on heuristic search algorithms, such as simulated annealing, tabu search, and genetic algorithm. Like sequential approaches that use enhanced data models, a triangulation-based data structure, simplicial data structure (SDS) was used in these approaches to detect conflict [10], [29]. However, these approaches evaluate the quality of a displacement solution merely on the basis of minimum distances between map objects. The complexity of the search for an optimal resolution increases considerably with the number of map objects and the trial positions. From a practical point of view, the effectiveness and efficiency of these approaches are unlikely to satisfy the requirements of displacement.

Function approaches are more reasonable than the combinatorial approaches. Function approaches model the problem of displacement as a global equation system based on certain methods developed in physics, mathematics, and/or engineering, such as energy minimization, finite element method (FEM), and least squares adjustment [7], [8], [30]–[38].

Based on the least squares adjustment method [32]–[36], a series of algorithms was proposed to resolve problems in generalization, such as simplification, displacement, aggregation, and typification. Map generalization was transformed into the problem of how to resolve optimally the situation with internal and external constraints. These constraints are expressed as linear functions of the object coordinates. Then, they are assembled into a design matrix such that the displacement problem is stated as a linear equation. To solve the equation system, the least squares method was used. Cartographic constraints can be formulated one-to-one in a system. However, the translation of ill-defined constraints into mathematical language is not always straightforward and requires further research.

The spring model may be the first energy minimization method for cartographic displacement in which various constraints on the line are modeled as different types of springs [30]. An “external potential” derived from the conflicts is used to trigger the displacement. Then, a balance between the “internal potential” based on the springs and the “external potential” is found using the downhill simplex method.

Using structural mechanics, Højholt modeled the displacement as a continuous deformation process on an elastic body [37]. In the model, the map space is discretized through CDT, and different stiffness and boundary conditions are allocated to triangles according to map generalization constraints. The FEM can be used to solve displacement and propagation problems holistically with good preservation of spatial relationships and patterns. However, the algorithm encounters problems with large deformations.

Burghardt and Meier proposed an approach to displace linear features using the snake model, a popular technique in the computer vision and pattern recognition fields [31]. A snake is an energy-minimizing spline controlled through the interplay between external and internal forces. Although this approach to the linear displacement problem is powerful, questions remain on how to use the method regarding the cartographic behavior [38].

To develop a real two-dimensional model of the displacement problem and retain the successful components of the snake technique, Bader proposed a new energy minimization method called elastic beams [7], [8]. This system treats the road network as an elastic beam structure based on structural mechanics. In structural mechanics, the behavior of a beam under load consists of two characteristics: compression or stretching in the direction of the longitudinal axis and bending in the direction perpendicular to the principal axis. This description of stretching and bending provides a better steering mechanism for deformation of linear features in cartographic displacement. To resolve the building displacement problem, Bader designed an auxiliary structure called ductile truss. This truss is based on enriched minimum spanning tree and it represents and preserves important spatial patterns of building clusters [8]. In the ductile truss, buildings can be displaced indirectly using the elastic beam algorithm. In this study, a fully automated building displacement algorithm was produced by using a combination of CDT skeleton and elastic beams. Meanwhile, several improvements to the elastic beam algorithm are proposed.

### Constraints, Conflicts, and Measures Involved in Building Displacement Problem

Cartographic constraints define spatial and human requirements that a map or a map feature should fulfill [39], [40]. The constraint-based approach is a shared method used to control and evaluate the automated generalization operations [41]. In this study, buildings are assumed to be rigid bodies trapped within separate partitions during the displacement process. Therefore, certain constraints should be considered.

#### Legibility constraint

To render the map symbols legible, minimum distances between buildings and between buildings and surrounding streets (or other linear objects) must be confirmed. For instance, the human eye has a dissolving power of approximately 0.2 mm at a reading distance of 30 mm [42].

#### Positional accuracy constraint

The display of individual buildings is restricted to large- and medium-scale maps not exceeding a scale of 1∶100,000. Based on these scales, the displacements should be limited within a specific range (e.g., <0.5 mm).

#### Global constraint of spatial relationships and patterns

On the basis of neighborhood models in urban morphology, the maps are partitioned for contextual generalization operations [43]. The global structures and density distributions of building partitions (e.g., enclaves, blocks, and superblocks) should be preserved to the greatest extent possible.

#### Local constraint of spatial relationships and patterns

In each partition, local spatial relations (topological, proximal, and directional relations) between neighboring map objects and characteristics and patterns of fundamental building groups based on Gestalt principles (e.g., alignments and arrangements of buildings) should be preserved to the greatest extent possible.

Constraint violations may cause conflicts. Conflicts or violations of constraints can be detected and evaluated by using one or more measures. A measure is a procedure used to compute measurements. Assessing the need for generalization and evaluation of the success of generalization are based on these measurements [19]. To detect proximity conflicts (violations of the legibility constraint) or accuracy conflicts (violation of the positional accuracy constraint), and to evaluate the degree of violation of the corresponding constraint, we should calculate the value of the associated measure (i.e., minimum distance between objects or maximum magnitude of displacement) and compare it with a target value that should be met for an optimal map at the target scale. However, measuring the spatial relationship and pattern constraint is not easy. Spatial relationships can be classified broadly into topological, proximal, and directional relationships between spatial objects [44]. These relationships build more complex spatial structures and patterns, such as linear and curvilinear alignments and grid-like clusters. To measure the violations of these global and local constraints of spatial relationships and patterns, the states of the corresponding relations and patterns before and after displacement should be described and compared. Unfortunately, most of these relations are ill-defined, and the related constraints cannot be easily expressed by scalar geometric measures. For this reason, enhanced data models (e.g., triangulations, Voronoi tessellations, and proximity graphs) should be constructed and used to identify, represent, and preserve the spatial relationships and patterns during the displacement process. Certain statistical measures (e.g., distribution of relative local densities) can be used to evaluate the final displacement results.

In summary, the building displacement mechanism was here triggered by detecting the proximity conflict and controlled using a set of constraints, which are monitored by corresponding measures or imposed using enhanced data models. The constraints from different views often seem to contradict each other. There is probably no perfect solution that can satisfy all of the constraints. The goal of the mechanism is to find the optimal compromise between these constraints. To produce such a mechanism, two key techniques were investigated in this study, CDT skeleton and elastic beam displacement algorithm. The details of these methods are presented in the subsequent section.

## Methods

The purpose of this work was to find an approach to detect and resolve proximity conflicts among a set of buildings and street segments because of the reduction of map scales. Before the displacement operation itself, the map data set was divided into separated partitions based on the “divide and conquer” principle. Then, the CDT skeleton and elastic beam techniques were employed to detect and resolve proximity conflicts in an iterative manner for each partition.

### Data set partitioning

First, the map area was divided into partitions. Buildings in a specific partition have no conflict with buildings or street segments in other partitions. Similar ideas have been presented in several studies in which road and river networks were used as frameworks to partition the map spaces [2], [4], [6], [8], [24].

**Figure 1:**
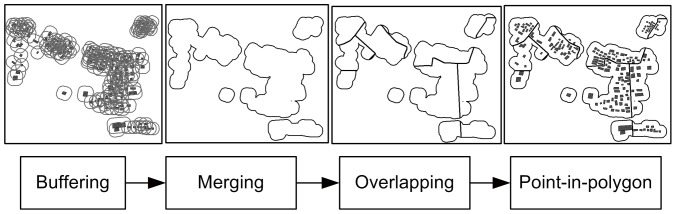
Data set partitioning. The data set partitioning method has four major steps: (1) buffering of buildings, (2) merging of buffering polygons, (3) conducting an overlap with street network, and (4) processing a point-in-polygon operation to assign each building to the corresponding partition.

A method proposed by Cecconi was used to derive feasible sub-partitions in both urban and suburban areas [17]. This method was originally used to matching process for buildings at different scales. The major steps of partitioning, as shown in Figure 1, are (1) conducting a buffering operation with suitable distance *r* (e.g., four times the positional accuracy threshold value), and assigning each building with a buffered area around it; (2) merging overlapping buffered areas into one/several large polygon/polygons that covers/cover all the buildings; (3) conducting an overlap with street network such that the large polygon/polygons derived in step (2) is/are divided into smaller ones, each being a partition; and (4) processing a point-in-polygon test (using the centroid as the representative point of each building).

### Extraction of CDT Skeleton

**Figure 2:**
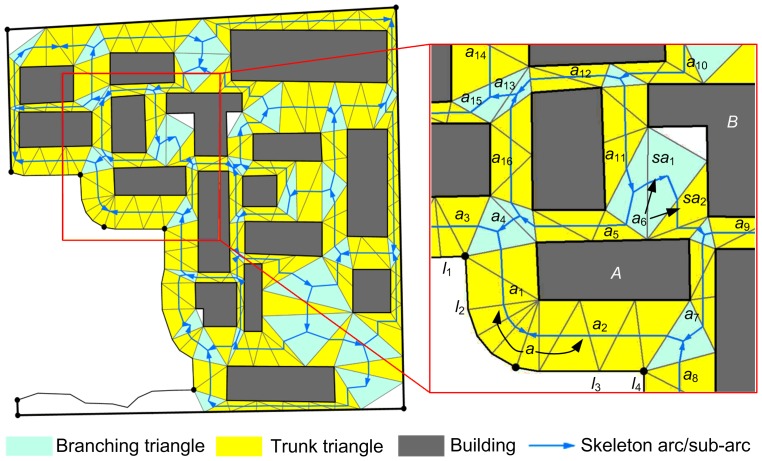
Extraction of the CDT skeleton. Extracted skeleton graph (left) in a map partition and detailed part (right) of the extracted skeleton graph to illustrate the concepts of skeleton arc, super-arc, and sub-arc.

To extract the CDT skeleton in a map partition, the CDT was constructed first. Given that the CDT construction algorithm had been investigated in the computational geometry community for many years [45], the present work focused on the process of skeleton extraction. To avoid generating extremely narrow triangles, additional points were inserted into some long constrained lines to refine the CDT [23], [34]. In the CDT, each gap space between a pair of map objects was covered by a sequence of triangles, known as a path of triangles. The path’s axis is called a skeleton arc. This arc can be extracted using the method proposed in [23]. Extracting and connecting all of the skeleton arcs produced a skeleton graph of the gap space of the partition (Figure 2).

However, the derived initial skeleton in this way may be not the final one. Two kinds of skeleton arcs, the super-arc and sub-arc, required a special process in the post-processing step. A super-arc is an arc that crosses more than one path and a sub-arc is a arc that crosses only part of a path. To establish a one-to-one correspondence between the skeleton arc and gap space (path), super-arcs were divided into segments (arcs) and sub-arcs crossing the same path were merged. As shown in Figure 2 (right), super-arc *a* was divided into two connected arcs *a_1_* and *a_2_*. Sub-arcs *sa_1_* and *sa_2_*, which are located at the same gap space between buildings *A* and *B*, were merged into arc *a_6_*.

### Construction and adjustment of the proximity graph

**Figure 3:**
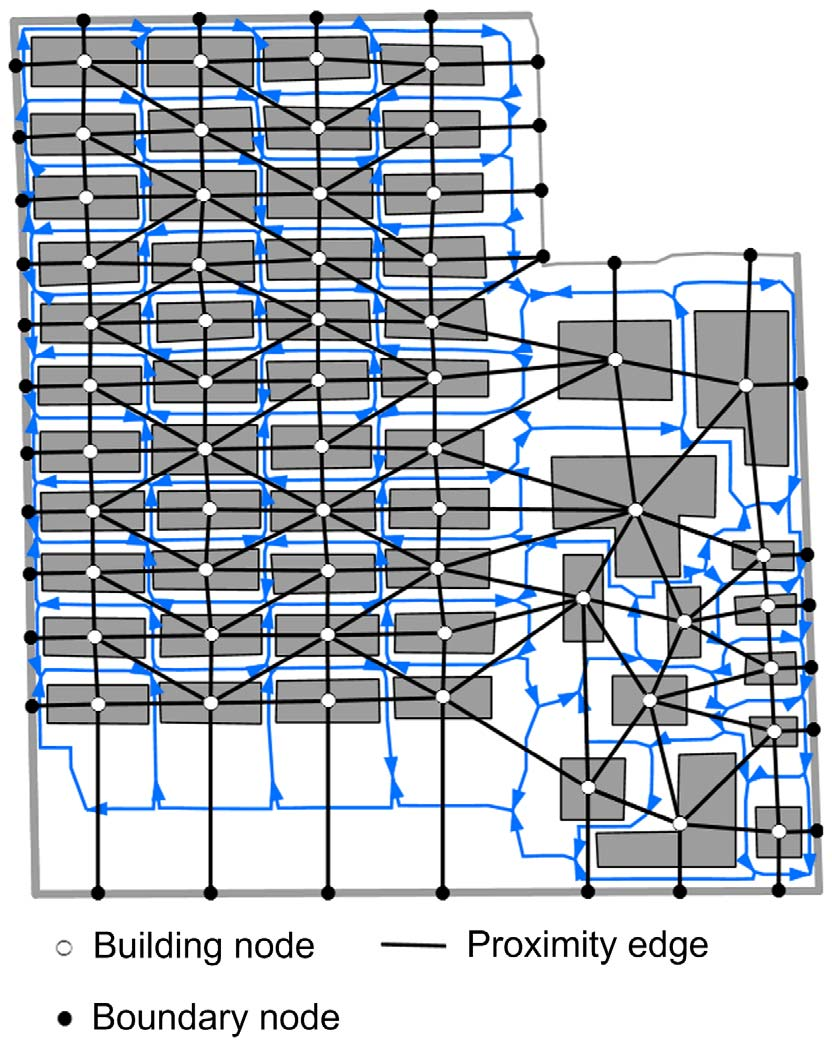
Construction of initial proximity graph from the skeleton graph. Each skeleton arc is corresponding to a proximity edge in the constructed proximity graph. There was a one-to-one correspondence between building and building node and a one-to-*n* (*n* ≧1) correspondence between boundary line segment and boundary node in the graph. The number of the boundary nodes on each boundary segment depends on the number of involved proximity relationships.

To enforce the constraints of spatial relations and patterns and to prepare for the use of the elastic beam algorithm, auxiliary structures should be constructed to represent the framework of the map partition, such as the ductile truss proposed in [8]. We use a kind of proximity graph that can be easily derived from the extracted skeleton graph (Figure 3). In the proximity graph, each node represents a map object, and each edge represents a proximal relationship between a pair of neighboring map objects. To reflect the geometric patterns of the map partition, the nodes of the graph were placed at some representative points of buildings and boundary segments. Concretely, a building node was located at the centroid of the border polygon of a building, and a boundary node (node on a street segment or a buffering boundary segment) was located at the point nearest the corresponding neighboring building node.

The proximity graph directly derived from the skeleton graph is a framework of the map partition. The global constraints of the spatial relationships and patterns can be enforced during the displacement process. However, certain local patterns of building groups based on Gestalt principles, such as collinear alignment, curvilinear alignment, and grid-like clusters, are also important characteristics and should be imposed as local constraints of spatial relationships and patterns [43]. Identifying these building groups remains a problem that is beyond the scope of this study. Building groups are here identified using the research results of previous studies [2], [43], [46]. These have been incorporated into the database as a type of supplemental information.

**Figure 4:**
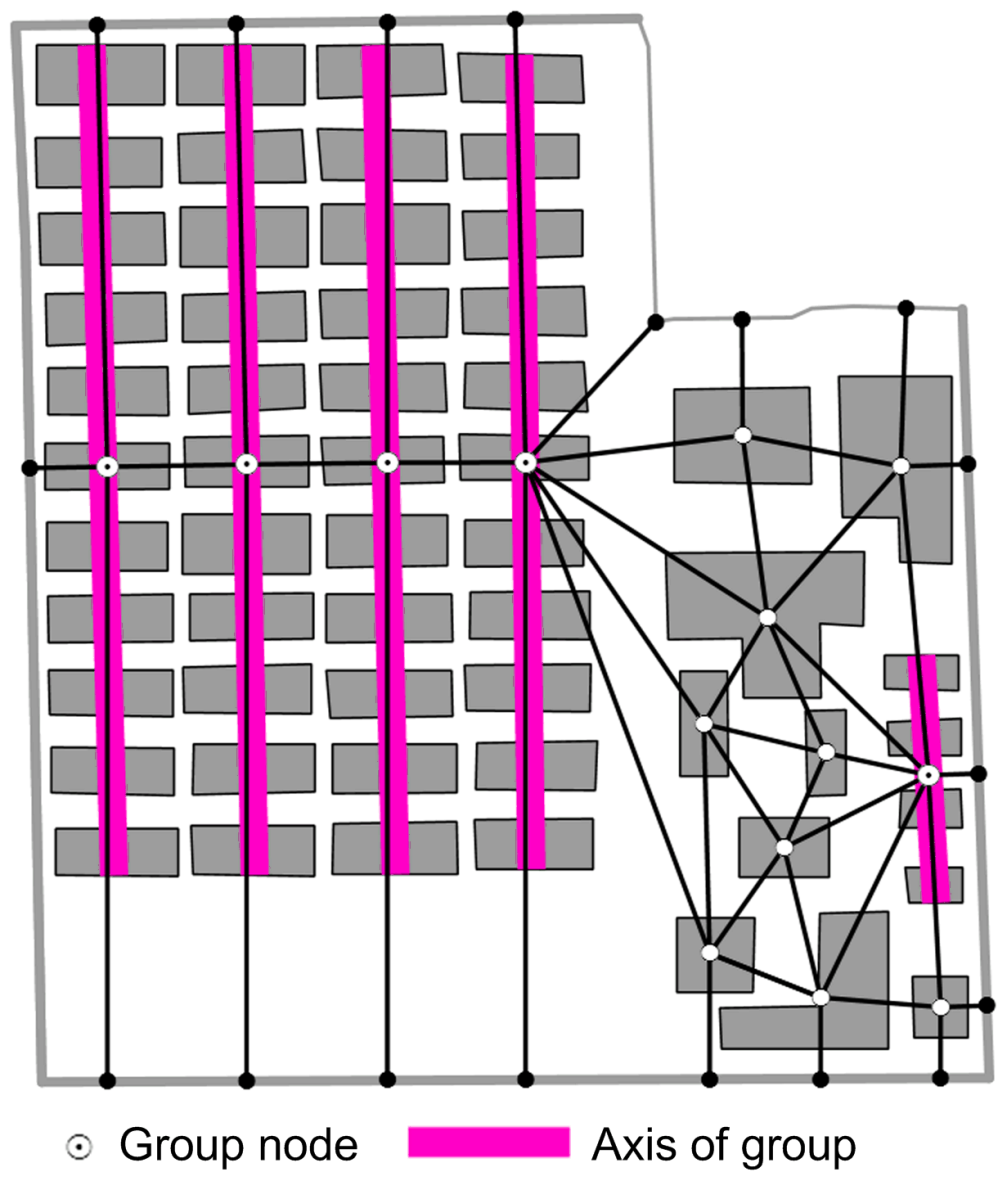
Adjusting proximity graph with building grouping information. Building groups are represented by group nodes in the adjusted proximity graph, and related proximity edges are deleted or collapsed.

We emphasize that the relative positions of the buildings in a local building group cannot be changed to preserve the local constraint of relationships and patterns unless there are conflicts within the group. If a group of buildings did not have any internal conflicts, it was considered as a whole. On this basis, the nodes of this group of buildings were merged into a single node. In this way, the group of buildings was shifted as a unit during the displacement process. As shown in Figure 4, there was a huge grid-like cluster of buildings (composed of 44 buildings, in 4 columns, and 11 rows). It was decomposed into four collinear alignments because of the proximity conflicts between the columns. Finally, each alignment was represented by a group node in the adjusted proximity graph.

### Detecting conflict and calculating external force

Cartographic displacement is triggered by proximity conflict, which is attributed to the inadequate gap space between neighboring objects. Gap spaces between neighboring objects must be identified and measured so that proximity conflicts can be detected. Proximal information of the skeleton arcs/sub-arcs allows easy confirmation of whether any conflicts exist between each pair of neighboring objects and allows measurement of the severity of the conflict.

**Figure 5:**
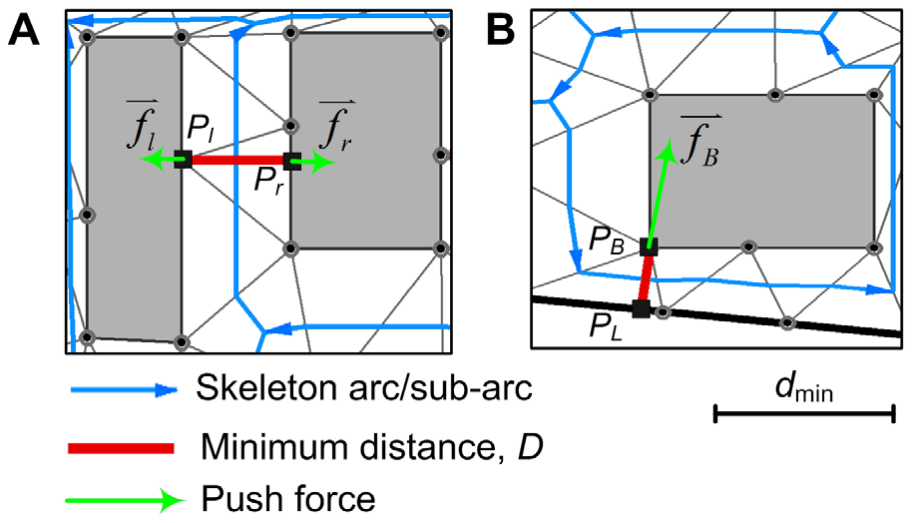
Detection of proximity conflict and calculation of push force. (A) BB-conflict and (B) BL-conflict.

Considering the sizes of map symbols, the minimum distance threshold between each pair of neighboring objects on the map is given by:
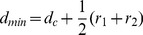
(1)where *d*_c_ is the minimum dimension threshold on maps (e.g. 0.2 mm), and *r*_1_ and *r*_2_ are symbol sizes of the two neighboring objects. Given a skeleton arc/sub-arc, the minimum distance between the left and right objects can be determined (Figure 5). If the minimum distance is less than the predefined threshold *d*_min_, a conflict exists. The severity of the conflict is given by:
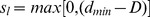
(2)where *D* is the minimum distance between the two objects.

In the energy minimization methods, the detected proximity conflicts gave rise to push forces that act upon conflicting buildings and trigger the displacement. In this case, two different types of conflicts were distinguished (Figure 5):

BB-conflict, which is the conflict between a pair of buildings.

BL-conflict, which is the conflict between a building and a street segment.

For the BB-conflict (Figure 5(A)), the push forces that act on the left and right buildings are calculated as follows:
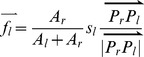
(3)




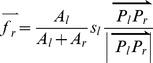
(4)where *A_l_* and *A_r_* are the areas of the border polygons of the left and right buildings, respectively; *P_l_* and *P_r_* are the two points with the minimal distance on the left and right buildings, respectively; and *s_l_* is the severity of the conflict.

For the BL-conflict (Figure 5(B)), the street segment is immovable; thus, the push force acting upon the building is given by:
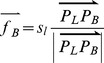
(5)where *P_L_* and *P_B_* are the two points with the minimum distance on the street segment and on the building, respectively; and *s_l_* is the severity of the conflict.

**Figure 6:**
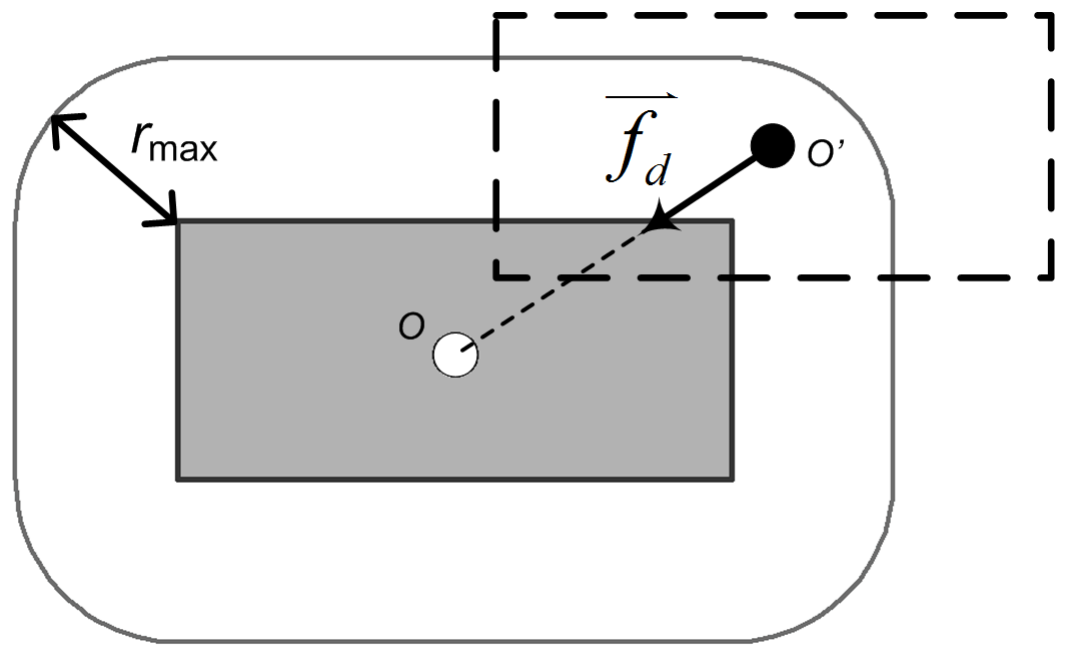
Positional accuracy constraint and drag force. *O* is the original position of the building’s centroid, *O′* is the new position of the building’s centroid after displacement, and *r*_max_ is the threshold of positional accuracy. If 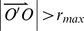, a drag force, 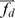, will be introduced to drag the building back to the acceptable area again.

In previous studies, the constraint of positional accuracy was not explicitly enforced [7], [8]. Positional accuracy cannot be assured. According to the specifications of topographic products, the magnitude of displacement should be limited within a threshold (e.g., 0.5 mm) [47]. The accuracy conflict was therefore defined to describe the violation of the positional accuracy constraint. For a building, once the displacement magnitude surpassed the positional accuracy threshold, an accuracy conflict occurs and its severity can expressed as follows (Figure 6):
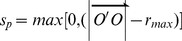
(6)where *O* is the original position of the building’s centroid, *O′* is the new position of the building’s centroid after displacement, and *r*_max_ is the threshold of positional accuracy.

Correspondingly, another kind of external force, drag force, was introduced to drag the buildings that violate the positional accuracy constraint back to the acceptable areas. For each building, the drag force is determined as follows (Figure 6):
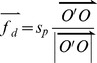
(7)where *s_p_* is the severity of the accuracy conflict.

When a building or group of buildings is affected by more than one individual external force (i.e., push and drag), we should combine these forces into a single resultant one. Calculating the resultant force directly through simple vector-based summing or averaging may cause inappropriate displacement. To avoid the accumulative effect because of the similarity of directions of some small forces, a more reasonable method may be used to combine the local maximum component forces in four main directions. The main idea is similar to that in [48].

### Using improved elastic beam algorithm

**Figure 7:**
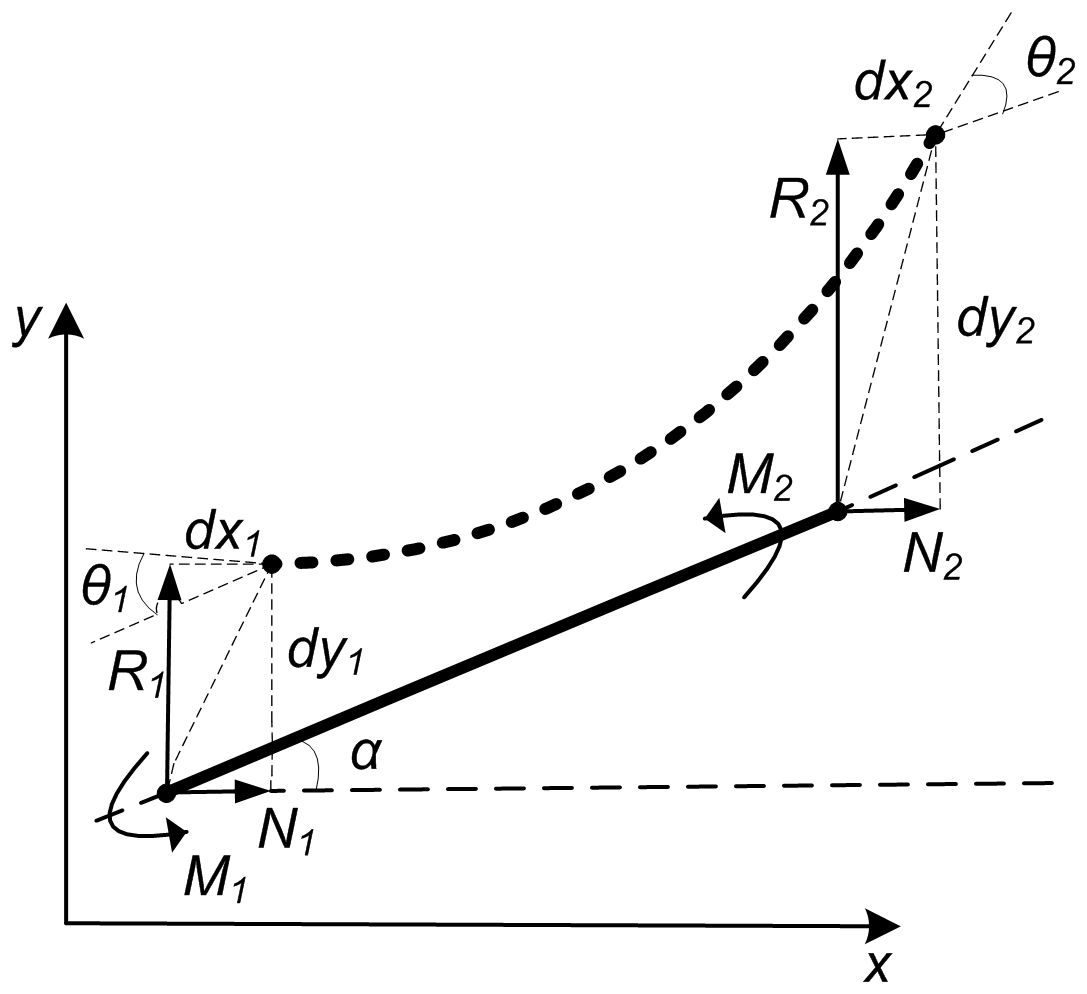
Force analysis of a beam element. 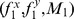
 and 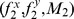 refer to the forces and moments applied to the end nodes, and 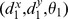 and 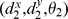 provide the displacements and rotations of the two end nodes.



FEM was used for the numerical resolution of the displacement problem described by the elastic beam model [7], [8]. Using FEM, the deformation solution of an individual beam was formulated as the following element matrix equation (for the detailed mathematical derivation process, see previous work [7]):
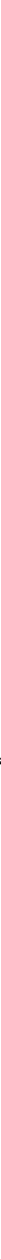
(8)where *l* is the beam’s length; 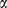 is the angle spanned by the beam axis and the x-axis, 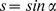 and 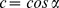, respectively; 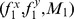 and 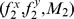 refer to the forces and moments applied to the two end nodes; and 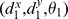 and 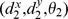 provide the displacements and rotations of the end nodes of the beam (Figure 7). In the element stiffness matrix, *E* is the Young’s modulus; *A* is the cross-sectional area; and *I* is the moment of inertia. The length of the beam and the moments on end nodes of the beam can be computed respectively as follows:

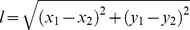
(9)

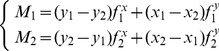
(10)where (*x*_1_, *y*_1_) and (*x*_2_, *y*_2_) are the coordinates of the beam’s two end nodes.

By integrating all of the element matrix equations, the following global equation system was determined:
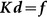
(11)where ***K*** is the global stiffness matrix, ***f*** is the force vector of equivalent computed shear and moment end actions (dependent on the external beam loading), and ***d*** is the displacement vector of unknown displacements and rotations of the beam ends.

Displacement is affected by the algorithm parameters (i.e., *E*, *A* and *I*) [7], [8]. Concretely, parameter *A* controls the stretching and compression of an element under axial load, and parameter *I* controls the bending behavior. By varying *A* and *I*, the susceptibility of the structure to bending and compression can be balanced. Parameter *E* is used to adjust the elasticity of beams. To take advantage of both the bending and compression properties of beams and to reduce the complexity of influence factors, we set *A* and *I* as constants that meet the condition *A≈I* (in our experiments *A* = 1 and *I* = 1). By contrast, the value of *E* was altered to adjust the scale of the magnitude of displacement under the action of external forces. Assuming that the maximum magnitude of displacement always corresponds to the maximum value of forces, a method of estimating the appropriate value of *E* by calculating the displacement twice is here proposed. In the first calculation, *E* was assigned as an arbitrary value E_0_ (>0). Then, the matrix equation (11) was solved to obtain the initial displacement vectors (displacement magnitudes and directions). The result of the first calculation is not feasible, but can be used to train the subsequent practical resolution. Using the derived initial displacement magnitude corresponding to the maximum force, the appropriate value of *E* can be estimated by using following empirical formula:
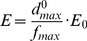
(12)where *f*_max_ is the maximum magnitude of external forces and 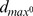 is the displacement distance corresponding to *f*_max_ in the first calculation. Then, using the estimated value of *E*, the displacement vectors is calculated again. In this way, the new displacement magnitude corresponding to the maximum external forces *d*_max_ can be almost equal to the maximum value of forces (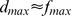). If the assumption is true, the building with the maximum force can be precisely displaced, and the remaining conflict can be resolved progressively in the successive iterations. Otherwise, the force may cause too many shifts in buildings. Nevertheless, a reasonable ratio between external and internal forces can be obtained from an overall view. This is essential to an energy minimization method. These inappropriate shifts can be corrected by drag forces in the successive iterations.

### Iterative strategy

Using displacement algorithm only once cannot guarantee that all conflicts will be resolved or no new conflicts will arise, particularly in complex situations. So the workflow of displacement is designed as a cyclic and iterative process that includes repeated conflict detection and resolution. Elastic beams produce a displacement solution when balance has been struck between external and internal forces. During the displacement process, the external forces become weaker when certain proximity conflicts have been resolved. The internal regularization forces continue to increase because of the shifting and deformation of map objects. When balance is struck, external and internal energy are not zero. This indicates that map objects may be still too close to each other in an equilibrium state. Therefore, the external energy should not be balanced with the inner one, but should vanish instead [7], [8]. For this reason, we used a different iterative strategy. The geometry of the elastic beams after the previous run was used as reference for the next run, resetting the inner energy back to zero. The iterative strategy can be expressed in matrix terms as follows:
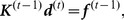
(13)where ***K*** is the stiffness matrix; ***d*** and ***f*** are the displacement and force vectors, respectively; and *t* is the current iteration time. Based on this iterative strategy, the overall process of conflict detection, displacement, and data set update for each partition is repeated as a cycle until the severity of conflict is reduced to an acceptable degree (e.g., the maximum severity of conflicts in the partition is reduced to 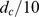) or the number of iterations is increased to a preset maximum number, *N* (e.g., 50) (Algorithm 1).

**Algorithm 1 Iterative strategy**

Input: the initial state of the map partition, ***P*** ^(0)^.

Initialize the iterative time, *t* ←1;

Repeat

Construct CDT and extract skeleton;

Detect conflict and compute force vector, ***f*** ^(*t*-1)^;

Construct and adjust proximity graph;

Set up algorithm parameter, *E*;

Compute global stiffness matrix, ***K*** ^(*t*-1)^;

Computer displacement vector, ***d*** ^(*t*)^ = (***K*** ^(*t*-1)^)^-1^ ***f*** ^(*t*-1)^;

Update coordinates of the displaced buildings;

*t* ←*t* +1;

Until maximum severity of conflicts, Max(*s)* <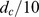 or *t>*50.

Output**:** the resultant state of the map partition, ***P*** ^(*t*)^.

## Results and Discussion

### Experimental results

The proposed approach was implemented in a testing generalization platform that had been developed based on the ArcGIS Engine developer kit. To validate the feasibility and adaptability of the approach, we chose two topographic data sets of different geographic areas (i.e., urban and suburban) with different scales (i.e., 1∶10,000 and 1∶25,000). Data set A is part of a data set of central urban areas, with a symbol width of 1.2 mm for the streets and an outline weight of 0.1 mm for the buildings (Dataset S1). Data set B is part of a data set of suburb areas, with a symbol width of 0.9 mm for the streets and an outline weight of 0.1 mm for the buildings (Dataset S2). For both data sets, the minimum dimension threshold on maps was 0.2 mm (*d*_c_ = 0.2 mm), the positional accuracy threshold was 0.5 mm (*r*_max_ = 0.5 mm), and the loop termination condition for iterations was formulized as maximum severity of conflicts <*d*_c_/10 OR number of iterations > 50. The tests were conducted using Windows on a 3.30 GHz Intel Core i3-2120 machine (2 Gb RAM).

**Figure 8:**
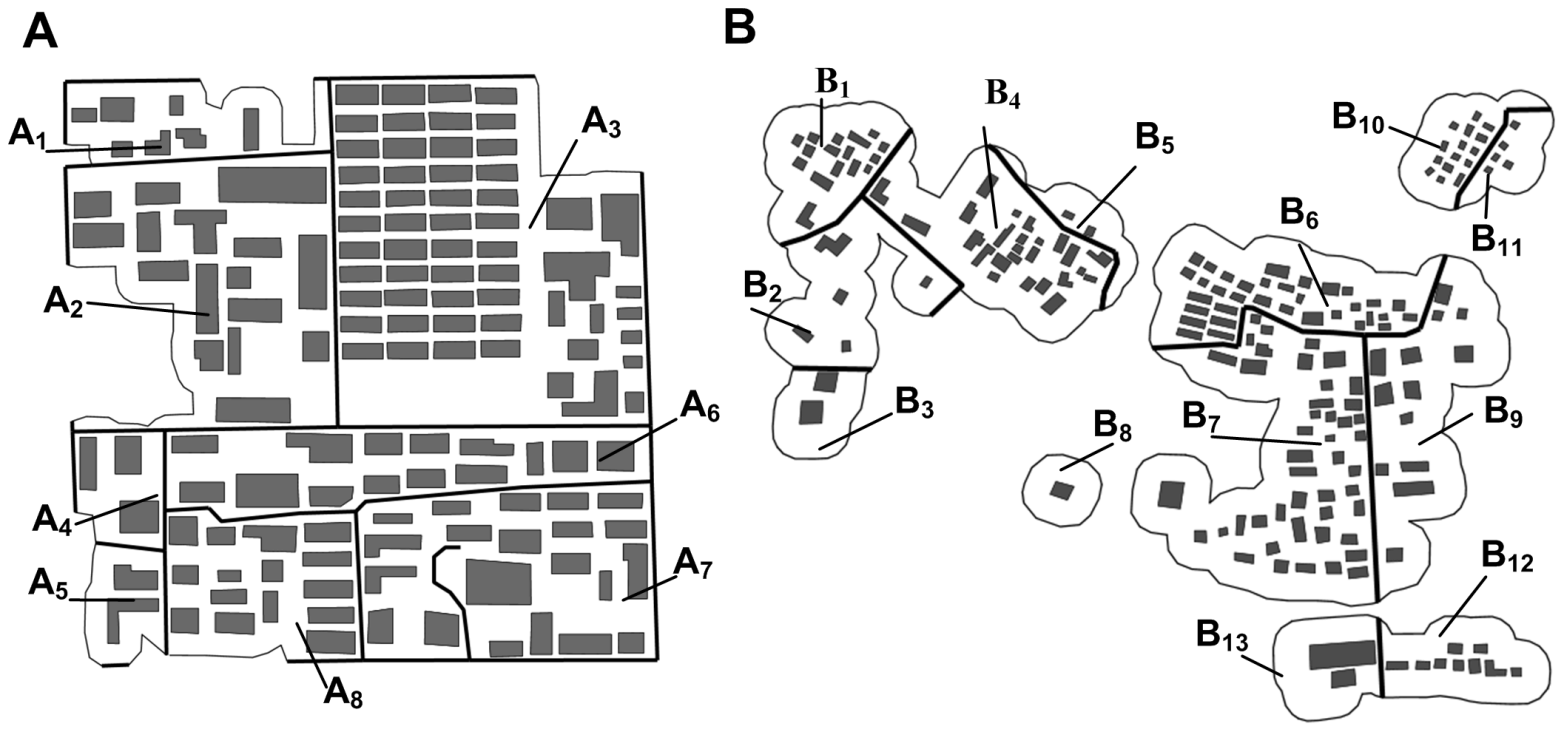
Testing data sets and partitions. (A) Partitions of data set A, and (B) partitions of data set B.

**Figure 9:**
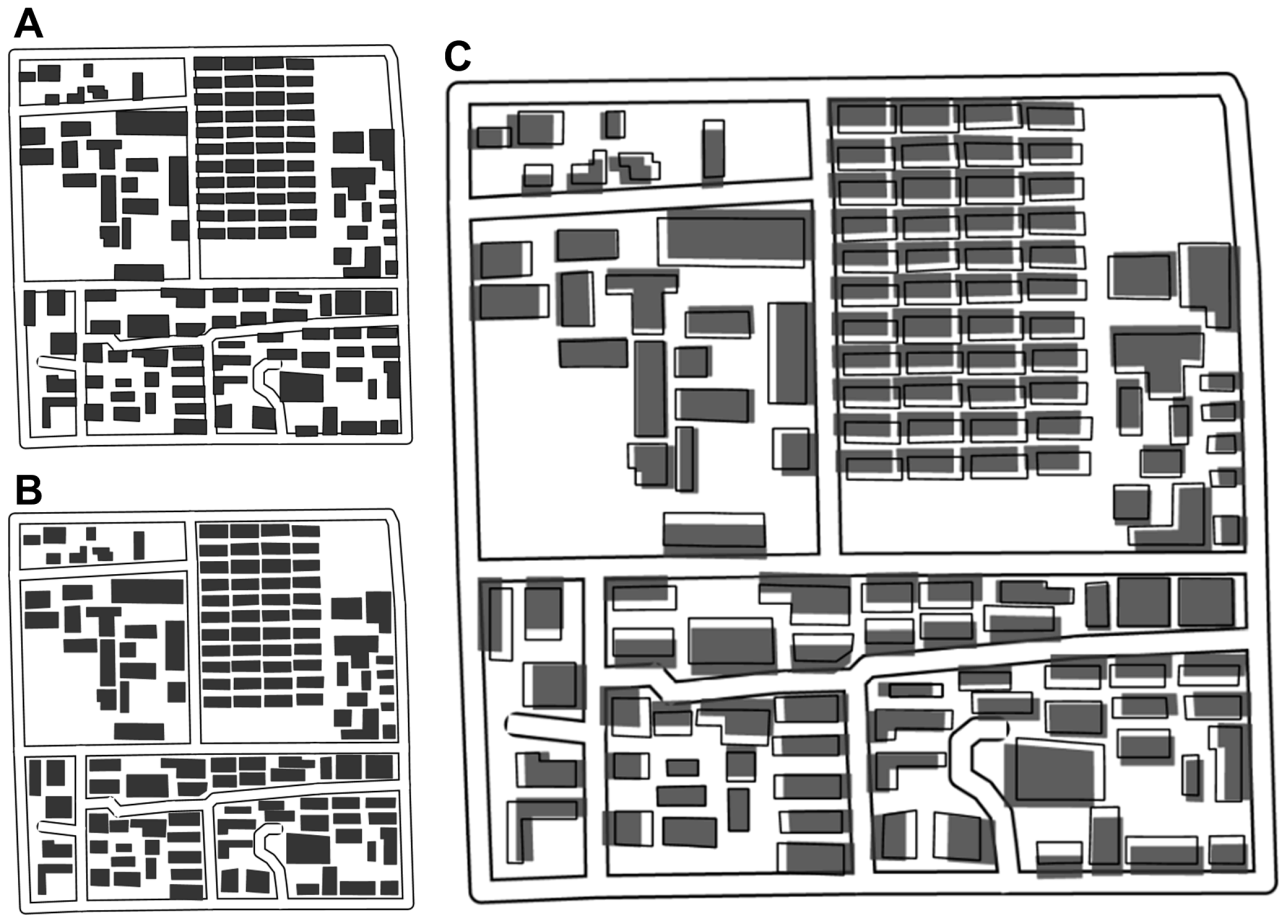
Visual result of data set A. (A) Initial situation (1∶10 000); (B) resultant situation (1∶10 000); (C) comparing the initial and final states (1∶10 000 enlarged to 1∶5 000).

**Figure 10:**
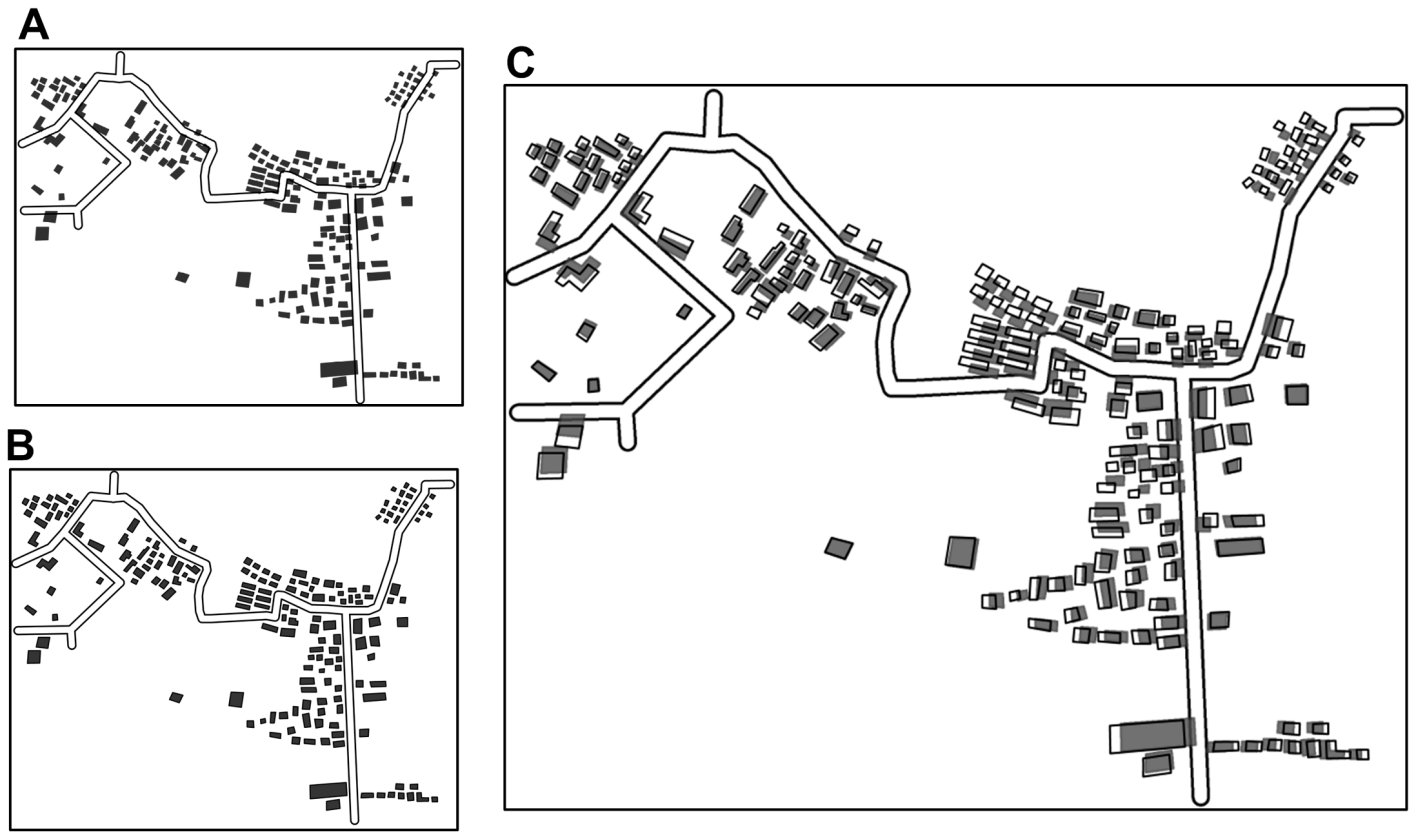
Visual result of data set B. (A) Initial situation (1∶25 000), (B) resultant situation (1∶25 000), and (C) comparing the initial and final states (1∶25 000 enlarged to 1∶12 500).

Figure 8 shows that the data sets were partitioned using the method described in the study, in which the radius for the buffering was 4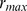. Figures 9 and 10 demonstrate the results and comparisons of the initial and final states for the two tests, respectively (for the intermediate results of the CDT, skeleton, proximity graph, and external forces, please see Figures S1–S4). From the perspective of visual effect, the resultant maps are significantly more legible than the original ones, and the spatial relationships and patterns of the buildings were well preserved. Detailed results of the experiments for each partition and total of each data set are shown in Tables 1 and 2, such as the count of initial and result conflicts, number of iterations and execution time.

**Table 1:** Results of data set A.

Partition	Buildings	Initial conflicts	Resultant conflicts	Iterations	Execution time (s)
A_1_	7	6	0	14	4.06
A_2_	16	7	0	5	1.76
A_3_	57	33	0	34	18.59
A_4_	3	5	0	4	0.94
A_5_	2	22	0	3	0.84
A_6_	14	16	6	50	23.4
A_7_	21	20	6	50	28.96
A_8_	15	13	5	50	12.9
Total	135	122	17	210	91.45

**Table 2:** Results of data set B.

Partition	Buildings	Initial conflicts	Resultant conflicts	Iterations	Execution time (s)
B_1_	16	9	0	38	15.3
B_2_	5	2	0	3	0.64
B_3_	2	1	0	2	0.31
B_4_	25	28	0	11	2.72
B_5_	2	2	0	2	0.37
B_6_	34	21	0	10	9.17
B_7_	45	22	0	32	74.77
B_8_	1	0	0	0	0
B_9_	13	6	0	18	4.11
B_10_	15	6	0	12	3.24
B_11_	5	4	0	3	0.55
B_12_	9	5	0	4	1
B_13_	2	2	0	2	0.39
Total	174	108	0	137	112.57

### Assessment of results

The purpose of the displacement is to ensure that the requirements of the related cartographic constraints are followed. Here a detailed assessment is presented with respect to each constraint.

#### Legibility constraint

The evaluation indicator of the legibility constraint is the number of proximity conflicts. Table 1 shows that, for data set A, the number of conflicts had reduced from 122 to 18 after displacement. The remaining conflicts were in partitions A_6_, A_7_, and A_8_. The spaces were extremely limited in the three partitions, and buildings clustered in specific limited spaces. Resolving the conflicts through displacement itself was impossible. The spaces of data set B were sufficient. The 108 conflicts in data set B were completely removed after displacement (Table 2). These results indicated that when the density of the buildings on the map is not very high, the approach could resolve all of the proximity conflicts.

#### Positional accuracy constraint

**Figure 11:**
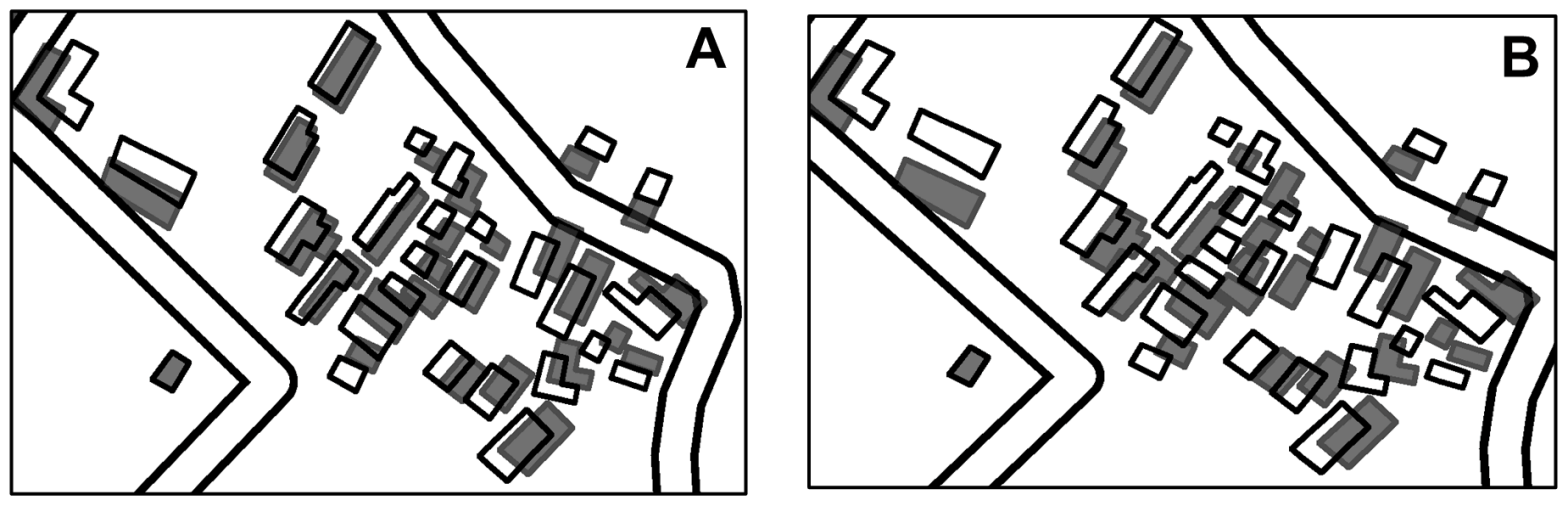
Comparative test on partition B_4_. (A) *r*_max_ = 0.5 mm and (B) *r*_max_ = 1.0 mm.

To preserve the spatial relationships and patterns for both data sets, almost all of the buildings were moved. This characteristic is a peculiarity of the elastic beam algorithm. However, statistical analysis of displacement magnitudes showed the maximum magnitude of displacement to be only slightly greater than the positional accuracy threshold (0.5 mm) (Table 3). The difference was acceptable because it was more precise than anything distinguishable by the human eye. The average magnitudes of the displacement of the two data sets were 0.39 and 0.35, respectively, which were both less than the threshold. In this approach, the positional accuracy constraint was enforced by introducing the drag forces. To prove the effectiveness of this enforcement, we conducted a comparative test on partition B_4_, in which the threshold of positional accuracy was 1.0 mm. The results indicated that the drag force is highly effective (Figure 11).

**Table 3:** Statistics of displacement magnitude for each data set.

Data set	Count(mm)	Maximum(mm)	Minimum(mm)	Mean(mm)	Sum(mm)	Standard deviation(mm)
A	135	0.56	0.07	0.39	53.22	0.10
B	173	0.56	0.05	0.33	56.86	0.11

#### Global constraint of spatial relationships and patterns

**Figure 12:**
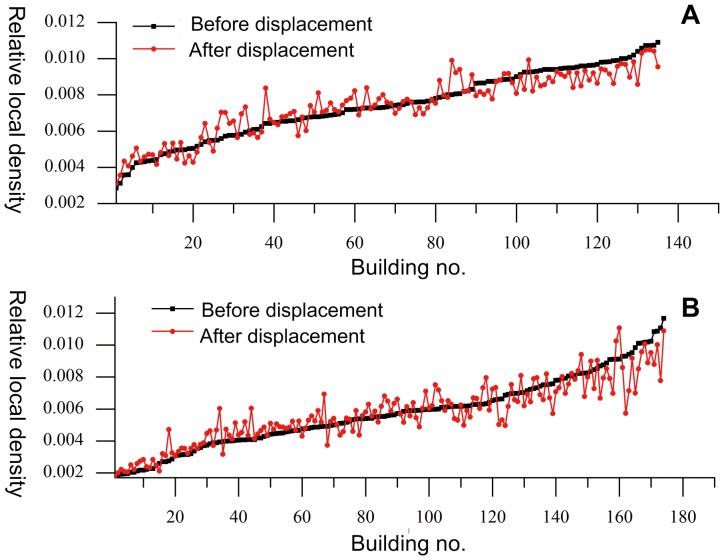
Curves of relative local densities. (A) Relative local density curves before and after displacement of data set A, and (B) relative local density curves before and after displacement of data set B.

To describe the extent to which global spatial relationships and patterns had been preserved, the density distributions of buildings was evaluated before and after the displacement. The relative local density of each building was used to express the density of each building over the entire region. To calculate the local density, a type of polygon tessellation which is highly similar to the VD was constructed based on the extracted skeleton graph (Figures S5 and S6). The diagram referred to here was a pseudo-Voronoi diagram (PVD). The relative local density of the *i*th building is given by [49]:
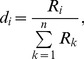
(14)where *d_i_* is the relative local density of the *i*th building, *n* is the number of buildings, and *R_i_* is the absolute local density of the *i*th building defined as follows:
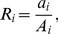
(15)where *a_i_* is the area of the border polygon of the *i*th building and *A_i_* is the area of the corresponding PVD polygon (for the relative local density of each building, please see Tables S1 and S2). To compare the change in relative local density, the curves for the relative local densities were drawn based on the strategy described in (Figure 12) [49]. As shown, the relative local densities before displacement were arranged in increasing order; and the relative local densities after displacement were arranged according to the same sequences (building no. means the corresponding sequence number). The overall trend of the relative local densities for both data sets remained the same, indicating that the density distributions of the buildings for both data sets are preserved well.

#### Local constraint of spatial relationships and patterns

**Figure 13:**
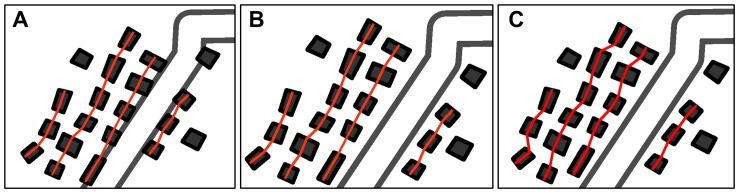
Comparative test on partitions B_10_ and B_11_. (A) Original state, (B) displacement result using proximity graphs with local grouping information, and (C) displacement result using proximity graphs without local grouping information.

In the adjusted proximity graph, the groups of buildings were treated as single units during the displacement. The relative positions of the buildings in each group were guaranteed to be 100% unchanged. To illustrate the effectiveness of this strategy, another comparative test was conducted on partitions B_10_ and B_11_ using proximity graphs without grouping information. Figure 13 showed that the building alignments in the test partitions were unchanged when the adjusted proximity graph was used (Figure 13(B)). The corresponding alignments were somewhat deformed, as indicated by the result of the comparative test (Figure 13(C)).

### Comparison to existing elastic beam algorithm

**Figure 14:**
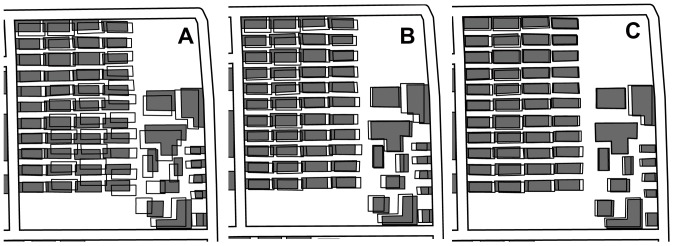
Comparative test results of existing elastic beam algorithm (partition A_3_). (A) *E* = 50 000, *A* = 1 and *I* = 1; (B) *E* = 200 000, *A* = 1 and *I* = 1; and (C) *E* = 1 000 000, *A* = 1 and *I* = 1.

We also implemented the existing elastic beam algorithm proposed by Bader et al. for comparison [7], [8]. In the comparative test, a suitable value of *E* was estimated for each partition through several rounds of trial and error. Due to space constraints, partition A_3_ served as an example. When *E* = 50,000, the buildings were displaced more than required; when *E* = 1,000,000, most of the conflicting buildings were still too close (proximity conflicts could not be resolved); and when *E* = 200,000, the result was similar with that of the improved algorithm, but there were still two proximity conflicts (Figure 14 and Table 4). The comparative test results show that the improvements on parameter-setting method and iterative strategy for elastic beam algorithm are effective and the current combined approach is more readily automated and feasible for conflict resolution.

**Table 4:** Statistics of the comparative test results (partition A_3_).

Test	*E*	Remaining conflicts	Maximum displacement (mm)	Mean displacement (mm)
A	50 000	0	0.95	0.63
B	200 000	2	0.54	0.4
C	1 000 000	28	0.31	0.26

### Potential usability and possible improvements

Displacement itself cannot guarantee the solution of all conflicts with insufficient map space available (Figure 9). For a complete solution of the conflicts in the graphic generalization process, other contextual generalization operators (e.g., deletion, exaggeration, aggregation, and typification) should be used and integrated with the proposed displacement approach. Implementation of other operators was not considered in this study, but the identified contextual information and resultant situations in these experiments provided useful information for the selection and operation of other generalization operators.

Map generalization also has considerable potential usability in some fields with good prospects such as location-based services (LBS) on mobile devices. Recently, using mobile visual search (MVS) and mobile visual location recognition (MVLR) techniques to enhance the user experience with respect to mobile devices has attracted a great deal of attention from researchers [50]–[55]. For this reason, the current approach should be integrated with these techniques in the future work. This would improve visualization effects of multi-scale geospatial data in LBS applications. However, to meet the requirements of mobile computing environments, the efficiency of our algorithm must be improved. For this purpose, the matrix sparsity methods [7] and parallel computing techniques [56] should be used.

## Conclusions

This study proposes a combined approach to automated building displacement in map generalization. Based on the combined use of elastic beams and CDT skeleton, the building displacement mechanism that is triggered by the detection of proximity conflicts and controlled by enforcement of a set of cartographic constraints was implemented using the proposed approach. The results of the tests showed that the implemented approach was feasible with respect to each constraint when the density on the map was not extremely high. The improved algorithm was found to be more readily automated and feasible for conflict resolution than the existing elastic beam algorithm [7], [8]. It also had more cartographic constraints. Future research aims to extend the implementation of our proposed approach by introducing other contextual generalization operators as well as to extend its field of application.

## Supporting Information

Figure S1 **Constructed CDT and extracted skeleton of data set A.**
(TIF)

Figure S2 **Constructed CDT and extracted skeleton of data set B.**
(TIF)

Figure S3 **Proximity graph and forces in data set A.**
(TIF)

Figure S4 **Proximity graph and forces in data set B.**
(TIF)

Figure S5 **PVD of data set A.**
(TIF)

Figure S6 **PVD of data set B.**
(TIF)

Table S1 **Density and displacement in data set A.**
(XLSX)

Table S2 **Density and displacement in data set B.**
(XLSX)

Dataset S1 **Data set A (before and after displacement).**
(RAR)

Dataset S2 **Data set B (before and after displacement).**
(ZIP)
