# Supplementary figures and images for: A Combined Approach to Cartographic Displacement for Buildings Based on Skeleton and Improved Elastic Beam Algorithm
Source: PLoS One. 2014 Dec 3;9(12):e113953. doi: 10.1371/journal.pone.0113953 (PMC4254966; doi:10.1371/journal.pone.0113953)

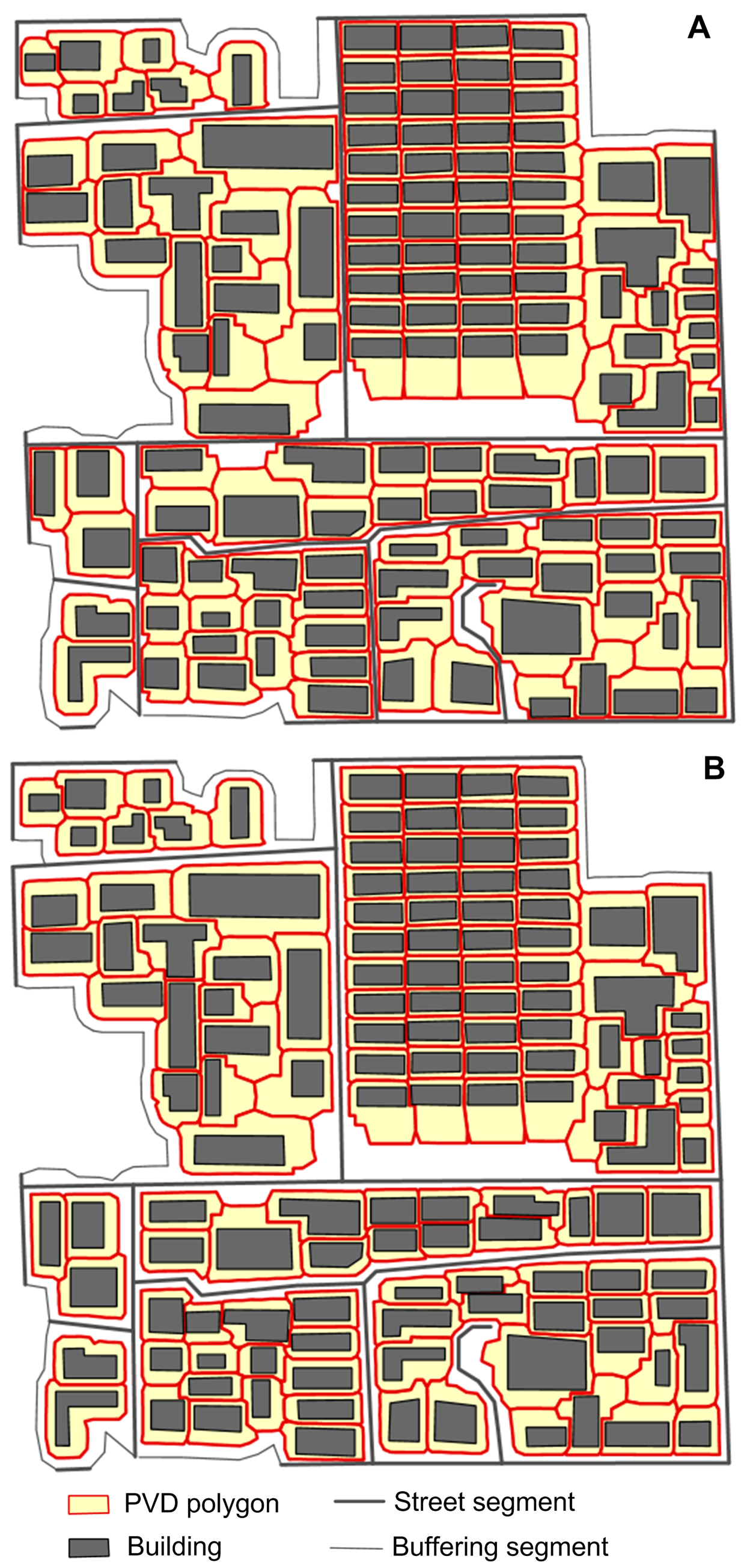

Supplement: Figure S5 — PVD of data set A. (TIF) [file pone.0113953.s005.tif]
